# Extracellular vesicles in idiopathic pulmonary fibrosis: pathogenesis and therapeutics

**DOI:** 10.1186/s41232-022-00210-0

**Published:** 2022-08-01

**Authors:** Yu Fujita

**Affiliations:** 1 Department of Translational Research for Exosomes, The Jikei University School of Medicine, 3-25-8 Nishi-Shimbashi, Minato-ku, Tokyo, 105-8461 Japan; 2 Division of Respiratory Diseases, Department of Internal Medicine, The Jikei University School of Medicine, 3-25-8 Nishi-Shimbashi, Minato-ku, Tokyo, 105-8461 Japan

**Keywords:** Extracellular vesicles, Exosomes, Idiopathic pulmonary fibrosis, Therapy

## Abstract

Idiopathic pulmonary fibrosis (IPF) is a progressive lung disease that occurs due to increased fibrosis of lung tissue in response to chronic injury of the epithelium. Therapeutic options for IPF remain limited as current therapies only function to decrease disease progression. Recently, extracellular vesicles (EVs), including exosomes and microvesicles, have been recognized as paracrine communicators through the component cargo. The population of cell-specific microRNAs and proteins present in EVs can regulate gene expressions of recipient cells, resulting in modulation of biological activities. EV cargoes reflect cell types and their physiological and pathological status of donor cells. Many current researches have highlighted the functions of EVs on the epithelial phenotype and fibroproliferative response in the pathogenesis of IPF. Furthermore, some native EVs could be used as a cell-free therapeutic approach for IPF as vehicles for drug delivery, given their intrinsic biocompatibility and specific target activity. EV-based therapies have been proposed as a new potential alternative to cell-based approaches. The advantage is that EVs, depending on their source, may be less immunogenic than their parental cells, likely due to a lower abundance of transmembrane proteins such as major histocompatibility complex (MHC) proteins on the surface. In the last decade, mesenchymal stem cell (MSC)-derived EVs have been rapidly developed as therapeutic products ready for clinical trials against various diseases. Considering EV functional complexity and heterogeneity, there is an urgent need to establish refined systemic standards for manufacturing processes and regulatory requirements of these medicines. This review highlights the EV-mediated cellular crosstalk involved in IPF pathogenesis and discusses the potential for EV-based therapeutics as a novel treatment modality for IPF.

## Introduction

Interstitial lung diseases (ILD) are a diverse group of rare and chronic lung disorders, with idiopathic pulmonary fibrosis (IPF) being the best-studied member. IPF is a progressive ILD that occurs due to increased fibrosis of lung tissue in response to chronic epithelial cell injury [1]. Increased extracellular matrix (ECM) replaces healthy lung tissue and destroys alveolar architecture, thus disrupting pulmonary compliance and eventually causing respiratory failure and death [2]. The features of IPF include a nonproductive cough and progressive dyspnea. Although the exact etiology of IPF development is still undissolved, the risk factors, such as tobacco exposure and dust, have been implicated. Five-year survival rate of IPF is worse than several types of cancer [3]. In general, the treatment includes oxygen supplementation, anti-fibrotic drugs, and lung transplant for severe disease. Regardless of the advances, therapeutic choices remain limited, as current treatment strategies only serve as decreasing disease progression. Furthermore, the strategies can be challenging as the IPF course is unpredictable due to the exacerbation episodes after a period of stability [4].

In the last decade, extracellular vesicles (EVs), including exosomes, microvesicles, and apoptotic bodies, have been recognized as a new paracrine mediator for the transfer of biological components [5, 6]. The cargoes of EVs mainly consist of microRNAs (miRNAs), proteins, and lipids that are transferred to recipient cells. EVs are emerging as novel cell-to-cell communication mediators and as possible biomarkers for various types of lung diseases [7, 8]. In fact, EVs detected in various biological fluids, including sputum, mucus, blood, urine, and bronchoalveolar lavage fluid (BALF), represent useful tools for both investigating the lung disease pathogenesis and for biomarker discovery. EV-derived compositions are an emerging therapeutic option for lung diseases. Some studies have shown that mesenchymal stem cell (MSC)-derived EVs can reverse the inflammation associated with chronic lung diseases [9]. In addition, EVs derived from specific cells or tissues have emerged as a novel cell-free modality for the treatment of IPF [10, 11].

In this review, I summarize current knowledge about EVs related to pulmonary fibrosis. I highlight the roles of EVs in the pathogenesis of IPF and their therapeutic potential for the condition.

## IPF pathogenesis and therapy

IPF is a chronic ILD characterized by the anomalous deposition of ECM in multifocal regions of the lung parenchyma [12]. Current research suggests that IPF arises as a result of repeated and persistent injury to the alveolar epithelium, which triggers signaling cascades by the immune system, resulting in chronic inflammation and finally fibrosis. The excessive inflammatory responses in IPF pathogenesis are thought to be multifactorial, and one of the key signals is transforming growth factor-ß (TGF-β) pathway. The mechanisms of chronic alveolar epithelial damage in IPF include various cellular processes such as cell death, cellular senescence, and genetic mutations [13].

Although IPF was first thought to be an inflammatory-driven disorder, many evidence and the failure of anti-inflammatory and immunosuppressive drugs in clinical setting have challenged this theory [14]. Indeed, corticosteroids and immunosuppressants have been used in the past, but at present, the recommendation is against the use of these agents for IPF based on the PANTHER-IPF trial [15]. In the latest update of the ATS/ERS/JRS/ALAT guidelines for IPF treatment published in 2015, only nintedanib and pirfenidone, along with antacids, are the medicines recommended for IPF treatment [16]. Pirfenidone, an inhibitor of TGF-β, and nintedanib, a tyrosine kinase inhibitor, were the key drugs to be approved by the Food and Drug Administration (FDA) and the European Medicines Agency (EMA) for the treatment. Both drugs have been shown to slow disease progression and prevent acute exacerbations of IPF [17–19]. Neither of these drugs are able to completely stop the IPF progression, and both are related to serious side effects. Current research efforts are focused on the development of novel therapeutics that target not only the aberrant deposition of ECM but also other signaling pathways including those mediated by host immune responses, as well as endogenous alveolar repair [20]. To cure chronic lung diseases including IPF, novel and innovative therapeutic approaches are needed.

## Extracellular vesicles

Almost all cells can secrete not only various types of soluble factors such as cytokines and chemokines, but also EVs into the extracellular environment. In general, EVs contain diverse components such as proteins, messenger RNAs (mRNAs), miRNAs, and lipids encapsulated in a phospholipid bilayer derived from either the endocytic compartments or plasma membrane of donor cells. EV components can be transferred to other cells, triggering a broad range of cellular signaling pathways and biological responses [5, 21, 22]. EVs are generally categorized as exosomes, microvesicles, and apoptotic bodies, based on their size, biogenesis, and secretory mechanisms (Table 1) [6]. Exosomes are small EVs (30–150 nm in diameter) and are released to the extracellular environment after fusion of late endosomes/multivesicular bodies (MVBs) with the plasma membrane. The MVB fusion with the plasma membrane is partially regulated by endosomal sorting complex required for transport (ESCRT), tumor susceptibility gene 101 protein (TSG101), ALG-2-interacting protein X (ALIX), neutral sphingomyelinase 2 (nSMase2), tetraspanins, Rab proteins, syntenin, and phospholipase D2 [23–25]. Microvesicles are medium/large EVs (100–1000 nm in diameter) and are generated by direct budding at the plasma membrane leading to direct release into the extracellular space. These EVs contain numerous proteins and lipids similar to those present in the membranes of the cells from which they originate [26, 27]. Most of the studies that deal with EVs have focused on exosomes and microvesicles, due to their various functions and implications. Apoptotic bodies are large EVs (>1000 nm in diameter) and are primarily produced by all cell types during the late stages of apoptosis [28]. However, current EV isolation methods cannot clearly distinguish between these different types of EVs based on size, origin, and density. Furthermore, EVs cannot be easily purified based on only protein markers on the vesicle membrane. Consequently, the minimal information for studies of extracellular vesicles (MISEV) guidelines published by the International Society for Extracellular Vesicles (ISEV) summarizes the difficulty in including biogenesis and origin as classification criteria for EV subtypes to work toward their wider acceptance and implementation [6]. In this perspective, it is important to follow the MISEV recommendations on EV research practice.

**Table 1:** Extracellular vesicle subcategories

EV subgroup	Size (nm)	Formation	Markers
Exosome (small EV)	30–150	Fusion of multivesicular bodies with the plasma membrane	ESCRT-associated proteins (Tetraspanins, ALIX, and TSG101)
Microvesicle (medium/large EV)	50–1000	Direct budding and cleavage of the plasma membrane	Cytoskeletal and plasma membrane proteins (origin cell-specific markers)
Apoptotic body (large EV)	>1000	Cytoplasmic fragmentation during programmed cell death	Proteins associated with the Golgi, endoplasmic reticulum, nucleus, and other cellular organelles.

EVs can transfer various important molecular messengers through cell-to-cell communication in terms of diverse physiological and pathological processes in recipient cells [21, 29]. There are some mechanisms by which EVs can interact with recipient cells to transfer their components. EV can release their cargoes via direct fusion with the plasma membrane, fusion with the endosome membrane, plasma membrane receptors, and endosomal rupture. After uptake by recipient cells, EVs can secrete their components into the cytoplasm of target cells, leading to modulation of specific signaling pathways. The cell-specific population of mRNAs and miRNAs present in EVs can alter the gene expression of recipient cells, resulting in the inhibition or activation of biological activities. In recent years, it has been demonstrated that EV-mediated genetic exchange between cells has elucidated novel important mechanisms in organ homeostasis and diseases pathogenesis. Quantitative and qualitative alterations in EVs may significantly contribute to the pathogenesis of diseases, including IPF. Moreover, recent works have focus on EVs for therapeutic medicine development as drug delivery vehicles given their intrinsic immunomodulatory and tropic activities [30].

## The roles of extracellular vesicles in IPF pathogenesis

EV components can be influenced by the physiological or pathological status of producing cells [31]. Although stem cell-derived EVs have the ability to repair and regenerate damaged tissues, EVs from specific cell types such as senescent cells and activated immune cells can induce pulmonary fibrosis [32]. Multiple studies have shown the emerging roles of EV cargoes in the epithelial-mesenchymal trophic unit during the development of pulmonary fibrosis.

Martin-Medina et al. identified WNT5A-positive EVs in BALF from IPF patients. They showed that secreted WNT proteins can be transported by EVs to promote lung fibroblast proliferation [33]. It has been demonstrated that the WNT signaling pathway has recently been implicated in the pathogenesis of various lung diseases including IPF [34]. WNT5A, a non-canonical WNT ligand, plays critical roles in stem cell renewal, cell migration, cell polarity, and inflammatory responses. The ligand is expressed in numerous cell types in the lungs, including lung epithelial cells and fibroblasts [35]. They found that lung fibroblasts are a major source of EV-bound WNT5A. This finding indicates that WNT5A on EVs isolated from IPF BALF leads to disease progression, highlighting the pathophysiological role of EVs in IPF.

Chanda et al. reported that senescent lung fibroblasts can induce fibroblast invasion through the direct interaction of EV fibronectin with recipient fibroblasts [36]. Fibronectin on the EV surface binds to integrin α5β1 and activates invasion signaling pathways involving focal adhesion kinase (FAK) and steroid receptor coactivator (Src) kinases. Together, this study uncovered a mechanistic insight of lung fibroblast invasion via EVs that may be relevant in the IPF pathogenesis.

Parimon et al. demonstrated that syndecan-1-positive EVs can promote pulmonary fibrosis by regulating epithelial reprogramming [37]. Syndecan-1 is a cell surface proteoglycan that is localized to the lung epithelium, placing it in an ideal location to govern lung mucosal immune responses [38, 39]. That study indicated that syndecan-1 controls epithelial cell plasticity and pulmonary fibrosis by altering EV miRNA profiles that can regulate fibrogenic signaling pathways, such as TGF-β and WNT signaling.

Kadota et al. showed that IPF lung fibroblast-derived EVs induce mitochondrial reactive oxygen species (mtROS) and associated mitochondrial damage to lung epithelial cells, resulting in mtROS-mediated activation of DNA damage responses and subsequent epithelial cell senescence [40]. The mechanistic study found that IPF lung fibroblast-derived EVs contain high levels of miRNA-23b-3p and miRNA-494-3p, which can directly suppress SIRT3 expression, leading to epithelial phenotypic changes. These data indicated a novel epithelial-mesenchymal interaction that is mediated by lung fibroblast-derived EVs during IPF pathogenesis.

Targeting the EV interactions in the epithelial-mesenchymal trophic unit may prove to be an effective therapeutic strategy for IPF. Furthermore, strategies for inhibiting specific EV protein/miRNA components or blocking the secretion of cell type-specific EVs might serve as new therapeutic options.

## Extracellular vesicle-based therapy for idiopathic pulmonary fibrosis

EVs have therapeutic potential as novel drug delivery vehicles. From a drug delivery perspective, EVs are biologically less likely to induce allergic immune responses and are considered to have a better safety profile than synthetic nanoscale carriers, such as liposomes and nanoparticles [41]. So far, some evidence suggested that native EVs containing endogenous anti-fibrotic components can be developed as natural therapeutic agents for pulmonary fibrosis. Furthermore, genetically engineered EVs with desired internal cargoes and specific targeting efficiency have better prospects for various types of disease treatment [42].

Currently, native EV-based therapeutic approaches for IPF are gradually being investigated. Previous reports have demonstrated that stem cell therapy as a therapeutic modality of regenerative medicine is a promising treatment strategy for various types of lung diseases, including IPF [43–46]. Current studies indicate that stem cell-derived paracrine effects are primarily responsible for this biological and therapeutic function of the cells [47]. Cell-free therapeutics have various advantages in overcoming the risks associated with cell use, such as immune incompatibility and embolization [48]. Therefore, cell-free EV-based therapeutics are emerging as a potentially efficacious alternative to cell-based therapy for regenerative medicine. In this regard, EVs possess distinct advantages including less immunogenic than their parental cells, likely due to a lower expression of transmembrane proteins such as major histocompatibility complex (MHC) compared with cell-based therapies [49]. Since stem cell-derived EVs naturally contain various bioactive molecules derived from stem cells, the EV-based therapeutics have several advantages in clinical therapies. Currently, various types of cell-derived EVs have been studied in experimental pulmonary fibrosis models, such as MSCs [50–54], adult stem cells [55], fibroblasts [56, 57], bronchial epithelial cells [10], and lung tissue spheroid cells [11] (Table 2).

**Table 2:** Extracellular vesicle-based therapeutics for pulmonary fibrosis models

Donor cells	Mouse models	EV delivery	Recipient cells	EV components	Ref
BM-MSCs	BLM	i.v	Alveolar macrophages	Unknown (modulation of monocyte phenotypes)	[50]
BM-MSCs	BLM	i.v	Lung fibroblasts	miR-29b-3p	[51]
BM-MSCs	BLM	i.v	Lung fibroblasts	miR-186	[52]
UC-MSCs	BLM	i.v	Alveolar epithelial cells, lung fibroblasts	miR-21-5p and -23-3p	[53]
UC-MSCs	silica	i.v	Lung fibroblasts	Unknown	[54]
Endometrial stem cells	BLM	i.v	Alveolar epithelial cells	*let-7*	[55]
Engineered dermal fibroblasts	BLM	i.t	Alveolar macrophages, lung fibroblasts, vascular endothelial cells	miRNAs with anti-inflammatory and anti-fibrotic functions	[56]
Bronchial epithelial cells	BLM	i.t	Lung fibroblasts, alveolar epithelial cells	miR-16, miR-26a, miR-26b, miR-141, miR-148a, and miR-200a	[10]
Lung spheroid cells	BLM/silica	i.h	Unknown	miR-30a, miR-99a, miR-100, and *let-7*	[11]

*BM-MSCs* bone marrow mesenchymal stem cells, *UC-MSCs* umbilical cord mesenchymal stem cells, *BLM* bleomycin, *i.v* intravenous, *i.t* intratracheal, *i.h* inhalation

MSCs are a class of multipotent cells that have a property of differentiation into a variety of cell types. MSCs can be isolated from multiple tissue sources, mainly bone marrow, adipose tissue, and umbilical cord tissue. In the last decade, the use of MSCs have emerged as a promising cell-based therapeutic strategy in the field of regenerative medicine. Furthermore, MSC-derived EVs have the potential to exert various effects such as immunomodulation and regeneration. Several preclinical studies have shown that MSC-derived EVs hold beneficial therapeutic effects for respiratory diseases such as IPF, chronic obstructive pulmonary disease (COPD), and acute respiratory distress syndrome (ARDS) [9, 58]. Indeed, Mansouri et al. demonstrated that human bone marrow (BM)-MSC-derived EVs can induce the anti-inflammatory phenotype of monocytes. The data demonstrated that human BM-MSC-derived EVs can alleviate pulmonary fibrosis and lung inflammation via lung monocyte modulation as a treatment for IPF [50]. Wan et al. also investigated human BM-MSC-derived EVs as a potential treatment for IPF. They showed that human BM-MSC-derived EVs with overexpressed miR-29b-3p could inhibit fibroblast proliferation by downregulating frizzled 6 (FZD6) [51]. Zhou et al. demonstrated that miR-186 packaged in human BM-MSC-derived EVs suppresses the expression of SRY-related HMG box transcription factor 4 (SOX4) and its downstream gene, Dickkopf-1 (DKK1), thereby blocking fibroblast activation and ameliorating pulmonary fibrosis. In vivo experiments using a bleomycin (BLM)-induced mouse model further showed that miR-186 released from human BM-MSC-derived EVs could suppress myofibroblast differentiation, indicating its therapeutic effects against IPF [52]. Shi et al. investigated the anti-fibrotic effect of human umbilical cord MSC (UC-MSC)-derived EVs using a BLM-induced mouse model. They demonstrated that human UC-MSC-derived EVs alleviate pulmonary fibrosis by repressing the TGF-β signaling pathway. Mechanistically, miR-21 and miR-23 in the EVs play crucial elements that contribute to anti-myofibroblast differentiation by downregulating TGF-β2 and TGF-βR2 expression [53]. While studies on MSC-derived EVs have dramatically increased, the development of clinical medicines is still in its infancy. One of the main challenges is in developing a scalable MSC-derived EV manufacturing paradigm. Recently, Xu et al. reported that EVs from 3D-cultured human UC-MSC spheroids have the potential to inhibit silica-induced pulmonary fibrosis and improve lung functions. They developed a novel method for a microcarrier-based bioreactor 3D system of UC-MSCs to continuously yield EVs. The 3D culture conditions may aid in the movement of nutrients toward the spheroids and waste products away from the spheroids, facilitating the viability of cells, thus yielding more UC-MSC-derived EVs than in 2D cultures within the same culture volume [54]. The culture system could expand MSCs for large-scale EV manufacturing in clinical settings.

As for different stem cell sources, Sun et al. demonstrated that human menstrual blood-derived endometrial stem cells (MenSCs), novel adult stem cells from human menstrual blood, relieve BLM-induced pulmonary fibrosis through their EV cargoes. Specifically, the EV miRNA *let-7* from MenSCs remits pulmonary fibrosis by modulating cellular reactive oxygen species, mitochondrial DNA damage, and NLR family pyrin domain containing 3 (NLRP3) inflammasome activation [55]. However, the therapeutic difference between multipotent stem cell- and adult stem cell-derived EVs for pulmonary fibrosis remains to be further studied.

Lung resident cells, including lung fibroblasts and bronchial epithelial cells, can be involved in airway homeostasis and have therapeutic potential for pulmonary fibrosis through their EV cargo [7]. For instance, Lacy et al. found that interleukin (IL)-1β-treated lung fibroblasts stimulate their EV release of prostaglandin E2 (PGE2), inhibiting both TGF-β-induced myofibroblast differentiation and excessive ECM deposition on naïve lung fibroblasts [57]. The inhibitory actions of EVs are dependent on fibroblast activation with IL-1β, a known inducer of the rate-limiting enzyme of PGE2 synthesis, cyclooxygenase-2 (COX2), and are abolished by the treatment of source fibroblasts with a COX-2 inhibitor. Accordingly, activated lung fibroblasts have communication with adjacent cells to inhibit pulmonary fibrosis and preserve lung homeostasis through EV cargo, indicating a novel EV-based therapeutic strategy for IPF patients. Moreover, their data also suggest that the impairment of this pathway may be involved in the development of pulmonary fibrosis. In addition, airway epithelial cell-derived EVs can be implicated in epithelial cell homeostasis in the lungs. Our recent study found that EVs derived from human bronchial epithelial cells (HBECs) can be ingested by epithelial cells and fibroblasts in airways and alveoli and inhibit epithelial cell senescence and myofibroblast differentiation via inhibition of TGF-β and WNT signaling pathways [10]. The therapeutic effect of HBEC-derived EVs is more pronounced than that observed with MSC-derived EVs. Highly enriched miRNAs in the EVs, miR-16, miR-26a, miR-26b, miR-141, miR-148a, and miR-200a, are mainly responsible for attenuating both myofibroblast differentiation and cellular senescence by blocking expression of WNT3A, WNT 5A, and WNT10B. Intratracheal administration of EVs in BLM-induced mouse model has attenuated pulmonary fibrosis development accompanied by decreased the expression of both β-catenin and cellular senescence markers. Crosstalk between the TGF-β superfamily and WNT signaling pathways plays substantial roles in the regulation of stem cell quiescence and activation [59, 60]. Through regulation of TGF-β/WNT signaling, HBEC-derived EVs could maintain lung homeostatic maintenance, repair, and regeneration [61].

As another EV source, Dinh et al. focused on the therapeutic potential of EVs derived from lung spheroid cells (LSCs) which contain a heterogeneous lung cell population [11]. Inhalation of LSC-derived EVs attenuated alveolar epithelial damage and ECM deposition in BLM- and silica-induced fibrosis models. Although the mechanistic insights remain to be fully elucidated, specific EV miRNAs such as miR-30a and the let-7 and miR-99 families may be partially involved in the attenuation of lung fibrosis and regeneration.

**Fig. 1:**
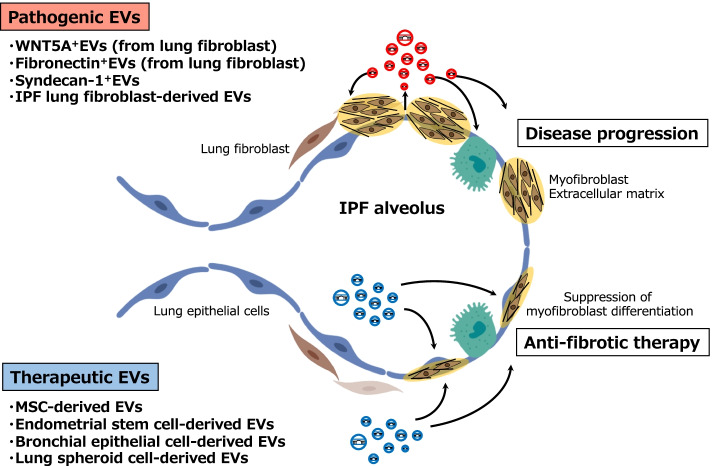
Extracellular vesicles in IPF: pathogenesis and therapeutics. In IPF lungs, pathogenic EVs mainly derived from lung fibroblasts can induce disease progression through their component cargoes, such as WNT5A, fibronectin, syndecan-1, or miRNAs. In contrast to this, therapeutic EVs derived from MSCs, endometrial stem cells, bronchial epithelial cells, or lung spheroids can inhibit myofibroblast differentiation and induce the degradation of extracellular matrix via shuttling of miRNAs

Despite the recent evidence providing that EVs have great therapeutic potential for IPF, further research should be performed to develop effective EV-based strategies. Although various types of stem cells are utilized for clinical applications in the field of regenerative medicine, MSCs are most commonly used stem cells due to their immunomodulatory capabilities and ease of successful isolation. In particular, future studies are needed to investigate whether MSCs or any of many other candidates, such as HBECs and LSCs, have the best relative potential for EV-based therapy in IPF patients (Fig. 1).

## Conclusions and future perspectives

Current studies indicate that EV-mediated cellular crosstalk is a novel regulator of pulmonary fibrosis. Notably, lung fibroblast-derived EVs can play key roles in IPF pathogenesis. For EV-based therapeutics, many pre-clinical studies have shown that MSC-derived EVs have potential therapeutic functions in respiratory diseases, including IPF. Importantly, the EV functions are dependent not only on the cell type but also on the microenvironmental changes, including cigarette smoke exposure, cytokines, and culture conditions. For instance, our group demonstrated that normal HBEC-derived EVs show anti-fibrotic properties by suppression of myofibroblast differentiation and cellular senescence [10]. On the other hand, EVs derived from cigarette smoke-exposed HBECs can induce myofibroblast differentiation associated with airway remodeling [62]. When designing a cell culturing process for EV production, it is essential to understand how culture conditions affect EV phenotypes. In fact, it has been reported that the MSC seeding density affects EV yield [63], and hypoxia has been shown to impact EV components such as proteins and RNAs [64]. Furthermore, emerging evidence suggests that cellular senescence may contribute to the dysregulation of EV components, resulting in the reduction of their therapeutic function [32]. During early-stage product development, the impact of cell quality control on EV functions must be well understood, as managing donor selection and the cell culturing process will likely be required for clinical settings.

Recently, MSC-derived EVs have shown rapid development as therapeutic products that are ready for clinical trials. So far, about 20 clinical trials of MSC-derived EVs as therapeutic interventions have been registered (listed in www.clinicaltrials.gov). For clinical-grade EV production, concentration by tangential flow-filtration (TFF) followed by size-exclusion chromatography (SEC) is becoming the preferred approach to better preserve the functional activity of EVs [65]. TFF is an efficient method for manufacturing EV population from large volumes of conditioned medium that are characterized by a rarefied EV content in reproducible manner. Combination of TFF and SEC allowed for the bilk processing of large starting volumes and the production of highly purified bioactive EVs in the clinical setting. Our knowledge regarding EV-based therapeutics is continuously expanding, but many questions remain unanswered for clinical applications. Several critical issues in EV-based therapy that need to be resolved are (i) scalable EV production methods, (ii) EV quantification and characterization, (iii) EV delivery route and pharmacokinetics, and (iv) safety profile assessments. Furthermore, it is essential to have appropriate identity and potency assays when studying EVs to make sure their quality control and reproducibility. Considering EV heterogeneity, there is an urgent necessity to develop refined systemic standards for manufacturing processes and regulatory requirements. This will enable the wider application of not only MSC-derived EVs, but also specific cell-derived EVs in IPF treatment trials. In addition, precise characterization of EVs is needed to reveal the EV-mediated mechanisms that underlie pulmonary fibrosis and for novel EV-based therapeutics. Their regenerative properties can also be utilized for other chronic lung diseases and organ fibrotic diseases, thus decreasing morbidity and mortality.

## Data Availability

Not applicable.
